# Unusual Case of Acute Intestinal Obstruction

**DOI:** 10.4021/jocmr439w

**Published:** 2010-10-11

**Authors:** Divey Manocha, Savio John, Nidhi Bansal, Manju Paul

**Affiliations:** a Department of Internal Medicine, SUNY Upstate Medical University, Syracuse, NY 13210, USA; b Department of Gastroenterology, SUNY Upstate Medical University, Syracuse, NY 13210, USA; c Department of Medicine, SUNY Upstate Medical University, Syracuse, NY 13210, USA; d Department of Pulmonary and Critical Care, SUNY Upstate Medical University, Syracuse, NY 13210, USA

## Abstract

Chronic NSAID use can cause diaphragm like strictures within the bowel leading to intestinal obstruction. This rare entity is called diaphragm disease. Preoperative diagnosis is extremely difficult. NSAID related injury should be considered in patient with intestinal obstruction of unclear etiology. We hereby present an interesting case of a 46 years old lady with significant history of long term naproxen use, who presented with nausea, vomiting and abdominal pain. She was diagnosed with acute intestinal obstruction. She received a trial of conservative management which was unsuccessful. Exploratory laparotomy with segmental resection of ileum was done subsequently. Histopathology showed mucosal ulceration, inflammation and focal sub mucosal fibrosis consistent with diaphragm disease. Patient recovered well in the post operative period.

**Keywords:**

NSAIDS; Acute Intestinal Obstruction; Diaphragm Disease

## Introduction

A NSAID-induced damage to the intestinal epithelium secondary to decreased prostaglandin and thrombaxane synthesis results from 3 phases of NSAID exposure [1, 2]. It includes preabsorption phase, local effects after direct exposure with oral administration, systemic effects after absorption, and recurrent local effects after enterohepatic recirculation [3, 4]. The relative damage from each of these phases is difficult to ascertain and it varies depending on the age, co-morbid conditions and the type and duration of NSAID exposure. The reports of diaphragm-like strictures of the right colon induced by indomethacin suppositories [3] and NSAID induced diaphragm disease arising in a bypassed ileal segment [4] support the theory of the systemic effect of NSAIDs in the causation of diaphragm disease.

## Case Report

Our patient is a 46 years old Caucasian lady who presented with right lower quadrant abdominal pain, nausea, vomiting and constipation for a week. There was no history of fever, hematochezia, recent travel, upper respiratory illness or sick contacts. Her past medical history was significant for chronic back ache of 20 years duration, controlled on Naproxen 200 mg twice a day as needed and methadone 10 mg three times a day. There was no history of previous abdominal surgery, inflammatory bowel disease or visceral cancers. On examination, she was tachycardic, normotensive, and afebrile. Cardiovascular and respiratory system examination was within normal limits. Examination of the abdomen showed no surgical scars. Bowel sounds were hypoactive. There was mild tenderness to palpation in the right lower quadrant with no peritoneal signs. Hernial orifices were intact and maneuvers for acute appendicitis were normal. Labs showed no evidence of leukocytosis, anemia or dyselectrolytemia.

Plain abdominal X ray showed multiple air fluid levels. Computed tomography (CT) abdomen showed dilated small bowel loops with transition point in distal ileum with mesenteric edema and stranding. The patient was kept on bowel rest with IV fluids and nasogastric decompression. Repeat abdominal films showed persistence of small bowel dilation. Subsequently she underwent explorative laparotomy and a segmental small intestinal resection with end to end anastomosis and appendectomy was done. The operative findings were significant for a distinctly demarcated segment of decompressed ileum, one foot proximal to the ileocecal valve, with no obvious serosal abnormalities. On histopathology, localized mucosal ulceration with marked acute and chronic inflammation was found. There was focal submucosal fibrosis with no granulomas or thickening of muscularis propria. The appendix was normal. Thus the final diagnosis of diaphragm disease secondary to chronic NASID use was made. The patient recovered well in post operative period and was discharged home on the fourth postoperative day.

## Discussion

Diaphragm disease is a term used to describe the appearance of multiple, thin diaphragm-like strictures within the bowel [5]. This entity was first named by Lang et al in 1988, who reported seven cases of diaphragm-like septa narrowing the intestinal lumen in patients taking NSAIDs [6]. A spectrum of patterns was found ranging from multiple pathognomonic ileal mucosal diaphragms to broad strictures, similar to those seen as a complication of potassium pill induced ulceration. The characteristic histologic abnormality is localized submucosal fibrosis. The inflammatory changes that accompany the lesions seem to be secondary to the underlying process and nonspecific. The reported overall prevalence ranges from 8.4% to 66%. Bjarnason et al used nonspecific radionuclide scanning to identify a 66% prevalence in patients who had been taking NSAIDs for longer than 6 months [7]. The pathogenesis of this condition is largely unknown although it has been suggested that damage to the mucosa, for example ulceration in any form, would cause fibrosis and scarring leading to contraction of the bowel wall and subsequent stenosis. Going et al proposed that circumferential ulceration could be the precursor of diaphragm diseases in the small intestine because multiple circumferential ulcers were noted in one of their patients who took indomethacin for 25 years [8]. In the healing phase, submucosal granulation tissue matures into collagenous scar tissue. These contracting rings of scar tissue ultimately form diaphragms by acting as drawstrings across the bowel lumen. Colonic strictures have more recently been reported [9]. Medications reported as associated with this disease include acetylsalicylic acid, indomethacin, diclofenac, phenylbutazone, piroxicam, and mefenamic acid [10]. In an attempt to decrease the detrimental effects of NSAIDs on the gastro duodenal mucosa, researchers have developed enteric coated and timed-release medications. However, sustained direct contact of NSAIDs with small-bowel mucosa and prolonged use of NSAIDs are both thought to contribute to an increase in small-bowel enteropathy [11]. Some reports support the postulation that the integrity of the mucosa is violated by the direct contact of the NSAID drug, leading to mucosal ulceration. The subsequent reparative process eventually leads to diaphragm-like strictures.

Preoperative diagnosis of diaphragm disease is extremely difficult. A complete medication history and a thorough small bowel examination during exploratory laparotomy are warranted as the lesions could be easily overlooked. Conditions causing short segment strictures and obstruction such as adhesions from prior surgery, appendicitis, gall stone ileus, Crohn’s disease, ileal tuberculosis, small intestinal tumors, radiation enteritis, potassium pill induced ulceration, celiac disease and ischemic bowel disease should be considered before entertaining a diagnosis of diaphragm disease. Definitive surgical treatment options include small bowel resection and strictureplasty [6].

## References

[1] Bjarnason I, Macpherson A (1989). The changing gastrointestinal side effect profile of non-steroidal anti-inflammatory drugs. A new approach for the prevention of a new problem. Scand J Gastroenterol Suppl.

[2] Kelly ME, McMahon LE, Jaroszewski DE, Yousfi MM, De Petris G, Swain JM (2005). Small-bowel diaphragm disease: seven surgical cases. Arch Surg.

[3] Hooker GD, Gregor JC, Ponich TP, McLarty TD (1996). Diaphragm-like strictures of the right colon induced by indomethacin suppositories: evidence of a systemic effect. Gastrointest Endosc.

[4] Monihan JM, Hensley SD, Sobin LH (1994). Nonsteroidal anti-inflammatory drug-induced diaphragm disease arising in a bypassed ileal segment. Am J Gastroenterol.

[5] Santolaria S, Cabezali R, Ortego J, Castiella T, Salinas JC, Lanas A (2001). Diaphragm disease of the small bowel: a case without apparent nonsteroidal antiinflammatory drug use. J Clin Gastroenterol.

[6] Lang J, Price AB, Levi AJ, Burke M, Gumpel JM, Bjarnason I (1988). Diaphragm disease: pathology of disease of the small intestine induced by non-steroidal anti-inflammatory drugs. J Clin Pathol.

[7] Bjarnason I, Pounder RE (1988). Non-steroidal anti-inflammatory drug-induced small intestinal inflammation in man. Recent Advances in Gastroenterology.

[8] Going JJ, Canvin J, Sturrock R (1993). Possible precursor of diaphragm disease in the small intestine. Lancet.

[9] Roche JC, Morris-Stiff G, Champ C, Williams GT, Lewis MH (2008). Colonic diaphragm disease without significant non-steroidal anti-inflammatory drug use: a case report. Cases J.

[10] Halter F, Weber B, Huber T, Eigenmann F, Frey MP, Ruchti C (1993). Diaphragm disease of the ascending colon. Association with sustained-release diclofenac. J Clin Gastroenterol.

[11] Davies NM, Saleh JY, Skjodt NM (2000). Detection and prevention of NSAID-induced enteropathy. J Pharm Pharm Sci.

